# *outbreaker2*: a modular platform for outbreak reconstruction

**DOI:** 10.1186/s12859-018-2330-z

**Published:** 2018-10-22

**Authors:** Finlay Campbell, Xavier Didelot, Rich Fitzjohn, Neil Ferguson, Anne Cori, Thibaut Jombart

**Affiliations:** MRC Centre for Outbreak Analysis and Modelling, Department of Infectious Disease Epidemiology, School of Public Health, Imperial College London, London, UK

**Keywords:** Transmission, Epidemics, Chain, Tree, Genomics, Software, MCMC, Bayesian, Likelihood

## Abstract

**Background:**

Reconstructing individual transmission events in an infectious disease outbreak can provide valuable information and help inform infection control policy. Recent years have seen considerable progress in the development of methodologies for reconstructing transmission chains using both epidemiological and genetic data. However, only a few of these methods have been implemented in software packages, and with little consideration for customisability and interoperability. Users are therefore limited to a small number of alternatives, incompatible tools with fixed functionality, or forced to develop their own algorithms at considerable personal effort.

**Results:**

Here we present *outbreaker2*, a flexible framework for outbreak reconstruction. This R package re-implements and extends the original model introduced with *outbreaker*, but most importantly also provides a modular platform allowing users to specify custom models within an optimised inferential framework. As a proof of concept, we implement the within-host evolutionary model introduced with *TransPhylo*, which is very distinct from the original genetic model in *outbreaker*, and demonstrate how even complex model results can be successfully included with minimal effort.

**Conclusions:**

*outbreaker2* provides a valuable starting point for future outbreak reconstruction tools, and represents a unifying platform that promotes customisability and interoperability. Implemented in the R software, *outbreaker2* joins a growing body of tools for outbreak analysis.

## Background

Determining ‘who infected whom’ during an outbreak can yield precious insights into transmission dynamics of an infectious disease and subsequently inform infection control policies. Transmission tree reconstruction has been used to specify the contribution of individual cases and locations to overall transmission [1], characterise heterogeneous infectiousness within outbreaks [2, 3], evaluate the impact of control measures on transmission intensity [4, 5] and identify transmission routes [6]. Consequently there exists significant interest in designing methodologies for the inference of transmission trees from outbreak data, including temporal data (e.g. date of symptom onset), contact data, pathogen whole genome sequences (WGS) and geographic locations.

A large number of studies have addressed this problem in recent years (Table 1) [6–15]. These approaches differ in multiple ways, including in their underlying epidemiological models (e.g. SIR [8, 11], SEIR [9, 12] or branching process models [7, 14, 15]) and genetic models (e.g. non-phylogenetic [7, 10, 12, 16] or phylogenetic models [11, 13–15]), as well as their ability to account for unobserved cases and multiple infectious introductions. This methodological diversity is beneficial, providing various theoretical frameworks for outbreak reconstruction in different epidemic scenarios.

**Table 1:** Studies on outbreak reconstruction and their availability as software

Study	Available as software	Study	Available as software
Cottam et al. [23]	✗	Numinnen et al. [24]	✗
Aldrin et al. [25]	✗	Hall et al. [11]	✓
Jombart et al. [26]	✓	Worby et al. [10]	✓
Ypma et al. [27]	✗	Lau et al. [12]	✗
Morelli et al. [28]	✗	Soubeyrand [29]	✗
Ypma et al. [6]	✗	De Maio et al. [13]	✓
Stadler et al. [30]	✓	Kenah et al. [31]	✗
Jombart et al. [7]	✓	Klinkenberg et al. [14]	✓
Didelot et al. [8]	✓	Worby et al. [32]	✗
Mollentze et al. [9]	✗	Didelot et al. [15]	✓

Unfortunately, the implementation of these methodologies in a user-friendly computational framework to encourage their use by the wider scientific community has so far remained limited. Primarily, a large proportion of the methods described in the literature is not available as readily useable software (Table 1), requiring the user either to directly modify the original code if it is available, or implement the algorithm themselves if not. Moreover, the existing software tools were developed in parallel with little consideration for interoperability, accepting similar types of data or outputting similar results in different formats. This results in unnecessary time spent preparing and formatting the data, and hinders effective comparison of results produced under different models. Finally, the current software are generally inflexible, with few options to specify algorithm behaviour without modifying the often complex source code (e.g. [7]). This ranges from simple implementation issues, such as being limited to specific distributions for priors [14, 15], to more fundamental restrictions on the inferential process itself, in that the underlying epidemiological and genetic models are hardcoded and not customisable by the user.

To address these issues, we have developed *outbreaker2*, a flexible software tool for outbreak reconstruction. *outbreaker2* exploits the fact that most transmission tree inference methods, though based on very different models, are generally implemented in a similar manner. Most consider the same data, namely WGS and some form of temporal data (i.e. dates of symptom onset and assumptions on the distribution of incubation and infectious periods [7, 9, 12, 14, 15], or explicitly defined exposure intervals [10, 13]). The majority are also implemented in a Bayesian framework, and therefore describe prior distributions on parameters and likelihood functions that evaluate the plausibility of a given parameter sets under specific transmission and evolutionary models. Unobserved data, including the transmission tree itself, times of infection and unobserved cases, are generally modelled using augmented data [17]. Finally most methods use a Markov Chain Monte-Carlo (MCMC) algorithm to derive samples from the posterior distributions, using often complex proposal functions to explore alternative transmission scenarios.

*outbreaker2* generalises this procedure and allows the user to implement their own models by specifying custom prior distributions, likelihood functions and movement functions, which are then employed within a wider inference framework. This enables sophisticated customisation of the algorithm with minimal effort by the user, allowing a greater focus on methodological developments rather than their implementation. Importantly, it also permits for different modules to be developed and easily combined, so that outbreak reconstruction approaches can be tailored to specific diseases and epidemiological contexts. *outbreaker2* is implemented as a package for the R software [18], as part of a larger toolkit for epidemics analysis developed under the R Epidemics Consortium (www.repidemicsconsortium.org). In the following, we explain the rationale of this implementation and illustrate its modularity using a simple case study.

## Implementation

**Fig. 1:**
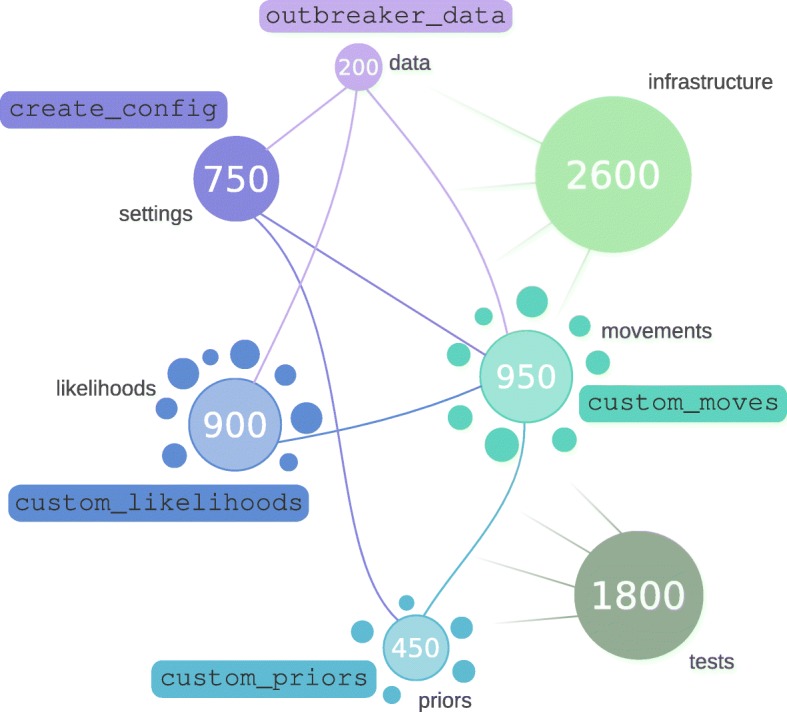
Schematic representation of the code design of *outbreaker2*. Each disk represents a different component of the code. Disk size matches the size of the corresponding component, indicated by numbers (in lines of code, rounded to 50). Separate disks for likelihoods, priors and movements indicate independent C++ modules. Links represent flows of information between components, colored according to the input. Infrastructure and tests are globally connected to all components. Functions indicated within rectangles are entry points into the code, indicating possible customisation by the user

*outbreaker2* is written in R and C++, making extensive use of Rcpp [19] to facilitate the integration of C++ into R. The original method for outbreak reconstruction introduced with *outbreaker* [7] has been entirely re-implemented using a modular and highly customisable approach (Fig. 1). This was achieved by distinguishing the architecture which underpins the inferential process from model-specific regions of code (i.e. code that varies between model implementations), and treating these components as independent modules (Fig. 1). Non-specific components of the implementation include data and overall configuration infrastructure, as well as all post-processing of outputs including summaries and graphics. The three central, model-specific components of our Bayesian inference framework are the prior distributions, likelihood functions and MCMC functions defining movements of the parameters. By abstracting these components into algorithmic functions with predefined input and output structures, we designed simple procedures allowing users to customise most aspects of the outbreak reconstruction, including the model itself and the MCMC used to explore the parameter space (Fig. 1). The following sections describes the structure of the core components of *outbreaker2*, and the mechanisms by which they can be changed. Extensive documentation, including a full description of the API and example of customised models, are available from the *outbreaker2* website (http://www.repidemicsconsortium.org/outbreaker2/).

### Object classes

*outbreaker2* defines several S3 object classes used to transfer information across modules. The outbreaker_data class stores the data which remains unchanged throughout the inference process. Users pass temporal data (e.g. sampling times) as a vector of dates, and genetic data as either DNA sequences (DNAbin objects [20]) or phylogenetic trees (phylo objects [20]). Generation time and incubation period distributions are also specified by the user. Extensive data validation is achieved by the constructor of this class to prevent often intractable errors at a later stage. The outbreaker_config class stores the global properties of the algorithm and can be optionally specified by the user. Importantly, this allows the user to declare which parameters and augmented data should be moved and inferred during the MCMC procedure. Again, the constructor of this class ensures validation of the inputs. The outbreaker_param class is used internally for storing a single state in the MCMC chain, and describes parameters and augmented data. Objects of this class are proposed, accepted or rejected, and sampled during the MCMC procedure. The advantage of this fixed internal structure is that it greatly simplifies writing new, customised movement functions. Finally, results of the reconstruction are output as outbreaker_chains objects, for which various methods (e.g. plot, print, summary) have been defined to summarise and visualise results, or carry on further secondary analyses.

### Custom prior distributions

A prior distribution describes the probability of observing a parameter given our previous knowledge of the infectious disease under observation. In *outbreaker2*, custom priors are specified as functions with a single argument of class outbreaker_param, which return a log-probability of a given parameter value (all probabilities are treated on a log scale). Custom priors can take any shape as long as this structure is satisfied. Priors can be specified for each parameter in the model by passing a named list of functions to the priors argument of the outbreaker function.

### Custom likelihood functions

Likelihood functions define the probability of a set of parameters (outbreaker_param object) given some observed data (outbreaker_data object) under a specific model. In *outbreaker2*, the overall likelihood is decomposed in separate likelihood components, which can be evaluated independently during the MCMC and therefore boost computer efficiency. Customised likelihood functions can be specified by the user for each of these components, to use alternative epidemiological or evolutionary models. Note that additional likelihood components can also be added, in which case the overall likelihood function will also need to be re-defined. Likelihood components can have any form, as long as they take an outbreaker_data and an outbreaker_param objects as arguments, and return a log-probability. As a result, users can combine different epidemiological and evolutionary models to fit specific needs.

### Custom movements functions

In an MCMC algorithm, movement functions are used to update the set of parameters and augmented data, from one MCMC iteration to the next. For example, a commonly used strategy is to use a Metropolis-Hastings move, where an update is first proposed and then accepted or rejected depending on its likelihood. Well designed movement functions are necessary to achieve efficient chain convergence and ensure rapid and representative sampling from the posterior distribution. In *outbreaker2*, the MCMC is decomposed as a list of movement functions, each of which is evaluated at each step of the chain.

Given the size and complexity of the parameter space when inferring temporally resolved transmission trees with unobserved cases, efficient movement functions are difficult and time-intensive to develop. *outbreaker2* allows users to access the optimised, default movement functions for various parameters and augmented data (including, crucially, the transmission tree) while using custom prior distributions and likelihood functions. Default movements, likelihood and prior functions can all be accessed through the function get_cpp_api, so that these components can be used when designing new MCMC procedures.

Unlike priors and likelihood functions which always take the same arguments, movement functions may have varying arguments including the data, general settings, custom priors and likelihoods. To simplify the specification of custom movements by the user, *outbreaker2* only requires that new movement functions have an outbreaker_param object for first argument; further arguments such as data and custom likelihood components are automatically detected, and internally replaced by the corresponding components of the code. In other words, the whole machinery of the code is added seamlessly to custom movement functions where it is needed. Importantly, as the acceptance-rejection step is specified within movement functions, users are not restricted to Metropolis-Hastings methods [21], and could use alternative MCMCs such as a Gibbs sampler [22].

## Results and discussion

### Implementing a custom model

The main asset of *outbreaker2* is its ability to define new models easily. We tested this flexibility by implementing the genetic model developed by Didelot et al. [8, 15] in the *TransPhylo* package. In contrast to the model of evolution used by *outbreaker2* [7], which treats mutations between all transmission pairs as independent events, *TransPhylo* uses a phylogenetic tree to account for patterns of common evolution amongst the sampled isolates. Briefly, *TransPhylo* takes a time-stamped phylogenetic tree as input, and explores ways of “coloring” this tree with one color for each infected host, thus revealing the evolution that occurred within this host. Transmission events are therefore also represented as the points of transition from one color to another, or in other words from one host to another. This approach is completely different from the one implemented in the default setting of *outbreaker2*, making it a good case study for the flexibility of implementation of custom models in the *outbreaker2* framework.

The genetic likelihood of *TransPhylo* was already implemented within the original package, and was therefore easily passed on to *outbreaker2* as a custom likelihood. To account for restrictions on the topology of the transmission tree in the *TransPhylo* model, a custom movement function on ancestries and infection times was also developed. This work was implemented in the R package *o2mod.TransPhylo* (standing for ‘*outbreaker2* module: *TransPhylo’*), which infers transmission trees using the *TransPhylo* genetic model while benefiting from the epidemiological model exploiting data on the incubation period and generation time distributions [7], extending the original Wallinga & Teunis model [5]. The total effort required to implement this model was minimal: only 185 lines of code (LOC) were necessary to design *o2mod.TransPhylo*, which is negligible compared to the 1434 LOC in the original *TransPhylo* package, or the 7633 LOC of *outbreaker2*.

We compared the performance of *o2mod.TransPhylo* and *TransPhylo* by reconstructing simulated outbreaks, using the simulator in the *phybreak* package described described by Klinkenberg et al. [14]. We used epidemiological and evolutionary parameters of Ebola virus as a plausible use case (Table 2), and assumed a linearly growing within-host pathogen population size. A total of 100 outbreaks each with 20 cases were simulated, and reconstructed using *o2mod.TransPhylo*, *TransPhylo*, and the default *outbreaker2* algorithm.

**Table 2:** Epidemiological and evolutionary parameters for Ebola virus. When several studies are cited, the mean value weighted by the sample size of the study was used

Parameter	Value	Reference
Generation time in days (SD)	14.4 (8.9)	[1, 33, 34]
Time-to-collection in days (SD)	14.4 (8.9)	Assumed same as generation time
Basic reproduction number R_0_	1.8	[33]
Mutation rate (per site per day)	3.1 × 10^− 6^	[35–37]
Genome length (bases)	18,958	[35, 38]

### Algorithm performance

**Fig. 2:**
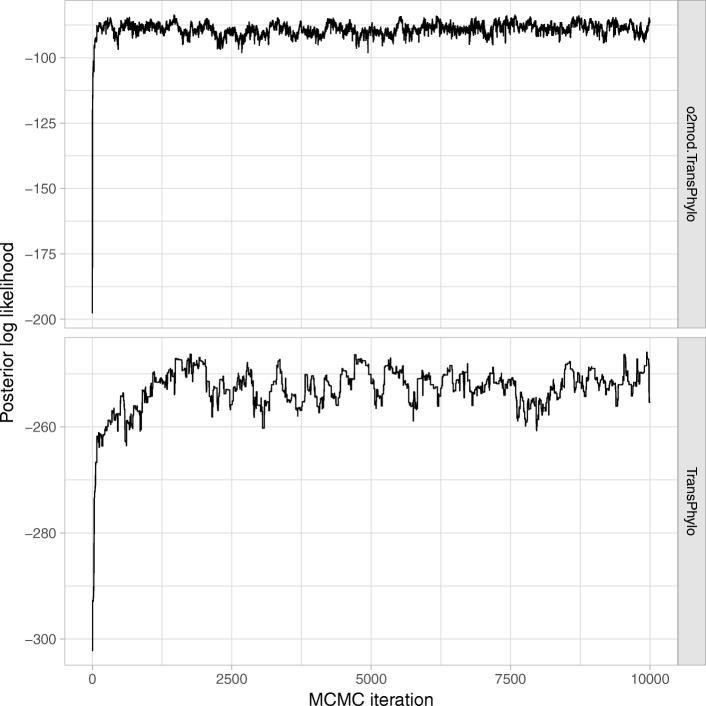
MCMC traces of posterior likelihood for *o2mod.TransPhylo* and the original *TransPhylo* package

MCMC chains of *o2mod.TransPhylo* converged rapidly, and mixed more efficiently than those of *TransPhylo* as demonstrated both by visual chain inspection (Fig. 2) and lower autocorrelation between log-likelihood values (0.31 and 0.72 at a lag of 50, respectively). This resulted in a significantly higher effective sample size per iteration (95.2 and 27.2 across a 5000 iteration window, respectively). However, individual iterations were significantly slower, taking on average 555.2 s per 1000 iterations, compared to 4.6 s for *TransPhylo* and 4.5 s for the basic model of *outbreaker2*. The vast majority of computational time by *o2mod.TransPhylo* was spent in the custom functions, which could be re-written in C++ for a performance boost. However, running times of *o2mod.TransPhylo* were acceptable for a complex Bayesian model: final results (well-mixed chain with 10,000 iterations) could be obtained in 1.5 h on a standard desktop computer. Users can therefore implement their models entirely in R and expect reasonable runtimes.

### Model outputs

**Fig. 3:**
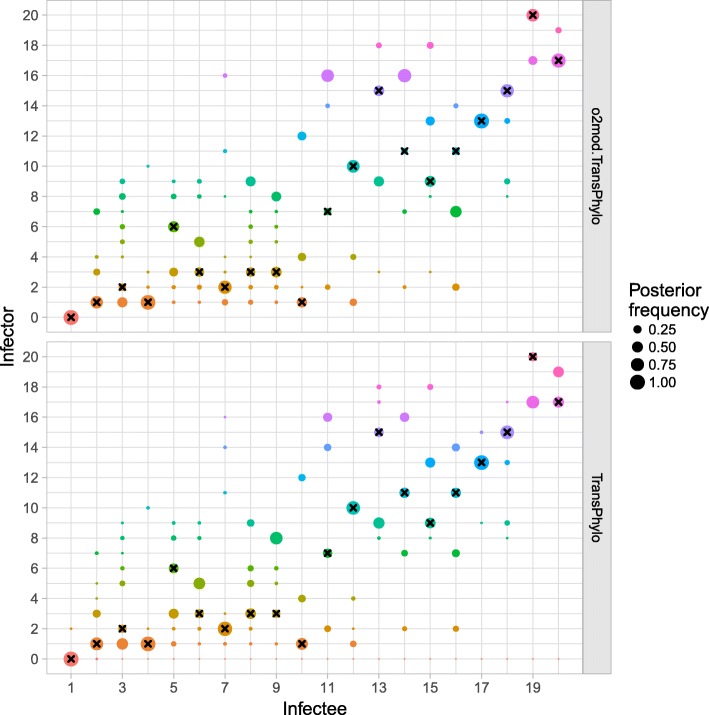
Posterior distribution of ancestry assignments using *o2mod.TransPhylo* and the original *TransPhylo* package. The size of each circle indicates the frequency of a given individual (“infector”) in the posterior distribution of infectors for a given case (“infectee”). An infector of 0 (bottom row) indicates that the individual is the index case. Black crosses represent the true simulated ancestries

Visual inspection of ancestry assignments for a single outbreak suggests that *outbreaker2* successfully explored the posterior distribution, and demonstrates that the inference framework is general enough to accommodate new models (Fig. 3). Encouragingly, *o2mod.TransPhylo* and *TransPhylo* appear to describe highly similar posterior distributions of ancestries, and agree on many assignments even if these have a very low posterior frequency.

**Fig. 4:**
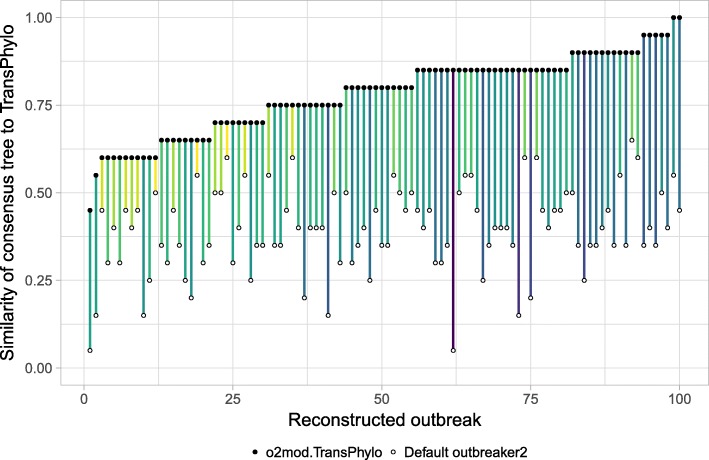
Similarity of consensus trees inferred by *o2mod.TransPhylo* and *outbreaker2* compared to *TransPhylo*. The consensus tree of a reconstructed outbreak is defined as the tree with the ancestor of the highest posterior probability for each case. The similarity between consensus trees is calculated as the proportion of identically assigned ancestries. The x-axis indicates individual simulated outbreaks. Each outbreak was reconstructed once using the default *outbreaker2* model (white dots), and once using *o2mod.TransPhylo* (black dots). The colour of the line represents the change in similarity to the consensus tree returned by the original *TransPhylo* package

To better compare the ancestry assignments made by *o2mod.TransPhylo* and *TransPhylo*, we used a consensus tree, defined as the tree with the highest posterior infector probability for each case, as a summary statistic. Across 100 outbreaks, on average 76.5% of ancestry assignments were equivalent. This represents a significant increase over the baseline similarity between the default *outbreaker2* model and *TransPhylo*, which agree on only 41.3% of ancestries on average, as confirmed by individual comparisons of consensus trees in reconstructed outbreaks (Fig. 4). It is important to note that 100% agreement between *o2mod.TransPhylo* and *TransPhylo* is not expected, as the epidemiological model of the latter parametrizes an offspring distribution and incorporates additional prior knowledge on its shape. However, the significant convergence in results upon using a custom likelihood acts as a proof of concept that *outbreaker2* can accurately recreate high level behaviour of largely different inference frameworks, and therefore represents a promising starting point for the implementation of future models.

### Future developments

*outbreaker2* is introduced as a flexible platform for outbreak reconstruction. We believe most future developments will occur through the creation of new modules by the community, distributed as separate R packages. Further adjustments may be made to accommodate additional epidemiological and evolutionary data and parameters currently not implemented in *outbreaker2*, and which may limit the scope for additional modules. Such changes will however be merely incremental, and should not represent any substantial development challenges.

## Conclusion

*outbreaker2* is a highly flexible outbreak reconstruction tool that can implement complex epidemiological and genetic models within an optimised and robust transmission tree inference framework. It allows users to focus on model development rather than software implementation, and provides a unifying platform for outbreak reconstruction tools that promotes interoperability and ease of use. We encourage the development of extensions to *outbreaker2* by the wider scientific community, with the goal of accumulating an extensive and sophisticated repertoire of methods for outbreak reconstruction within the R software.

## Availability and requirements

Project name: outbreaker2

Project home page: http://www.repidemicsconsortium.org/outbreaker2/

Project development page: https://github.com/reconhub/outbreaker2

Operating system(s): Platform independent

Programming language: R, C++

Other requirements: C++ 11

License: MIT

Any restrictions to use by non-academics: None

## Abbreviations

LOC: Lines of code
MCMC: Markov Chain Monte Carlo
